# Supplementary material for: Clinical Significance of Elevated Xanthine Dehydrogenase Levels and Hyperuricemia in Patients with Sepsis
Source: Int J Mol Sci. 2023 Sep 8;24(18):13857. doi: 10.3390/ijms241813857 (PMC10530551; doi:10.3390/ijms241813857)
Supplement: Supplementary file 1 [file ijms-24-13857-s001.zip › Supplementary figures.pptx]

## Slide 1
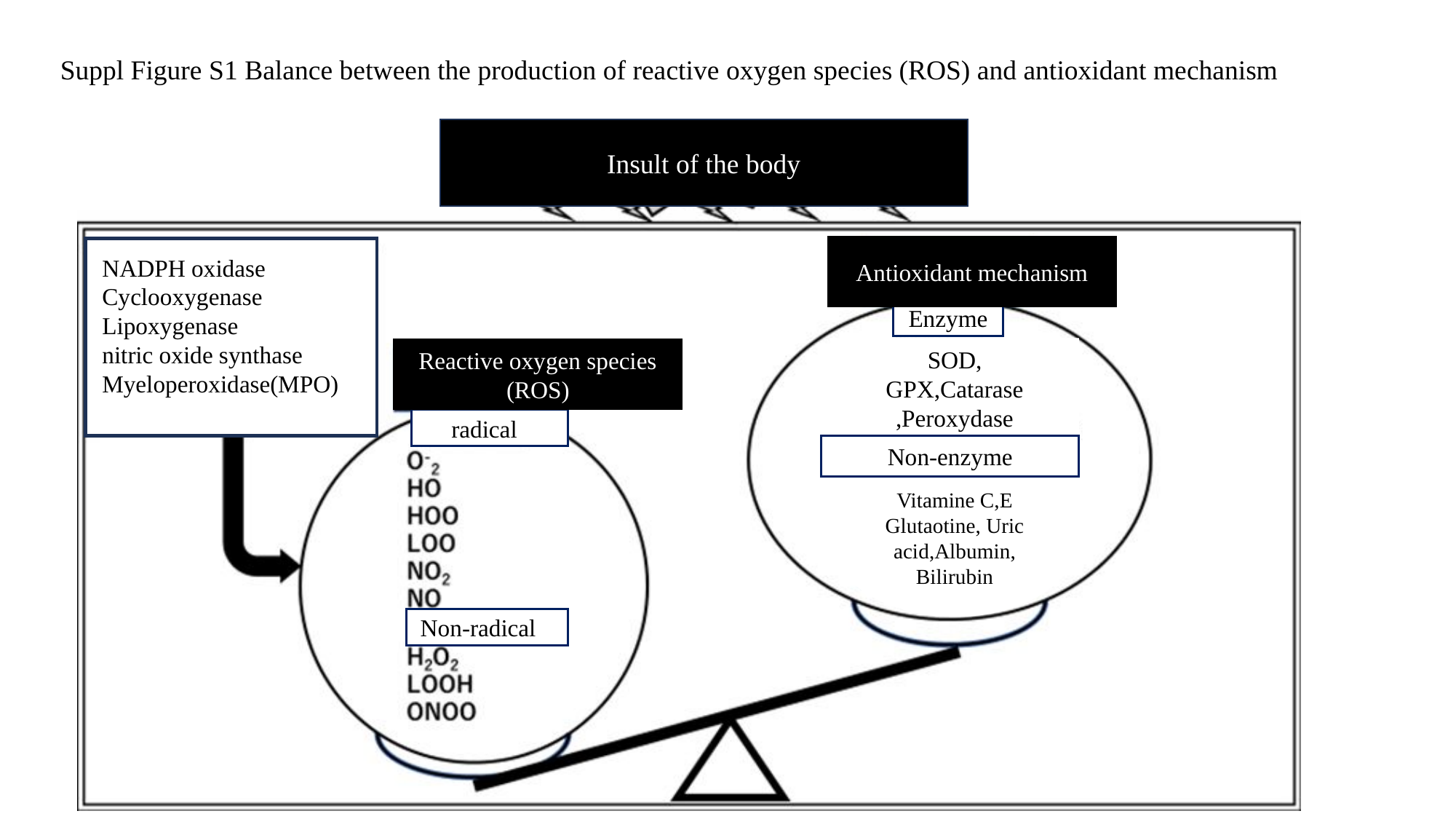

# Suppl Figure S1 Balance between the production of reactive oxygen species (ROS) and antioxidant mechanism
Insult of the body
Antioxidant mechanism
NADPH oxidase
Cyclooxygenase
Lipoxygenase
nitric oxide synthase
Myeloperoxidase(MPO)
Enzyme
SOD,
GPX,Catarase
,Peroxydase
Reactive oxygen species (ROS)
radical
Non-enzyme
Vitamine C,E
Glutaotine, Uric acid,Albumin, Bilirubin
Non-radical

## Slide 2
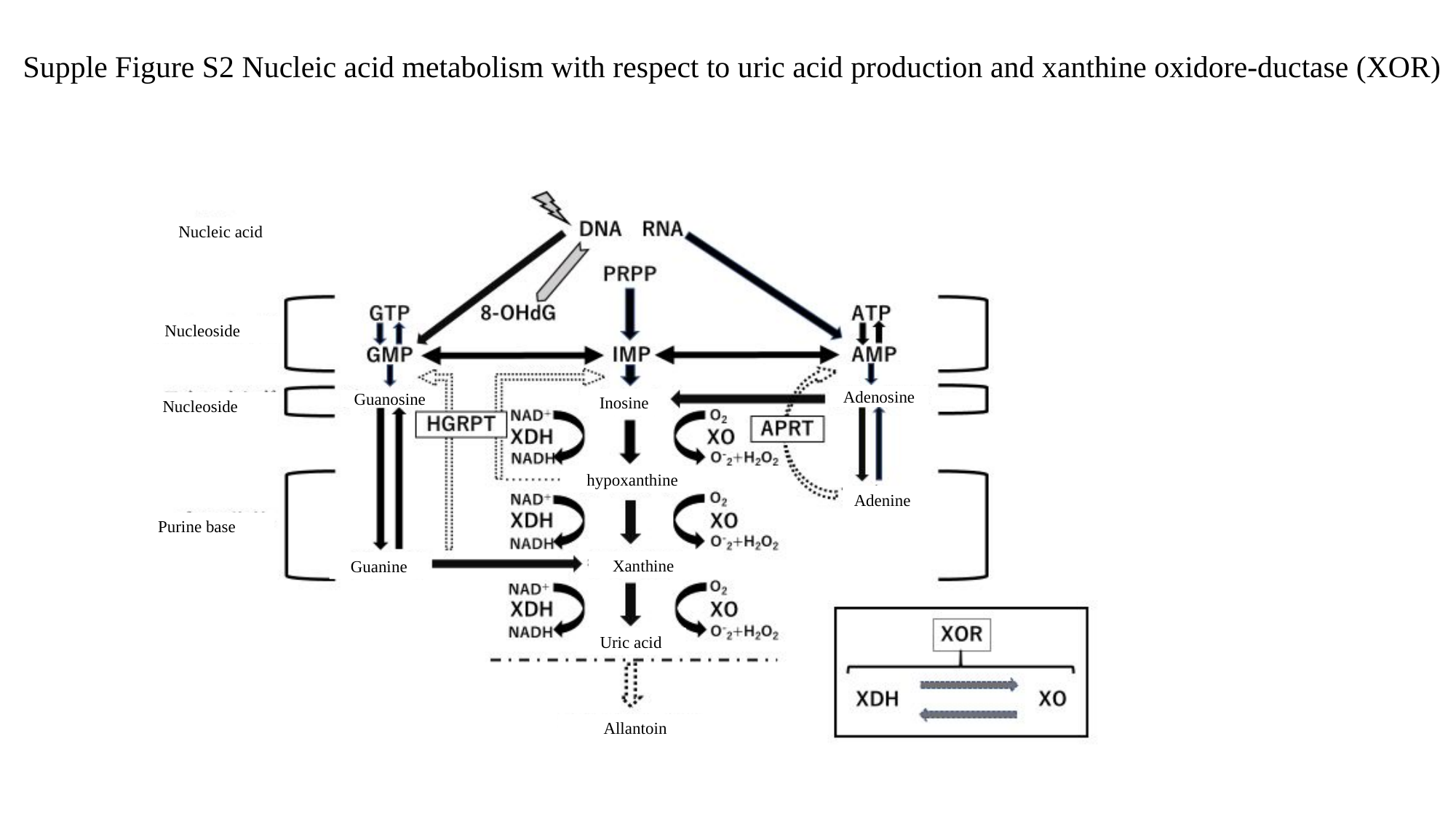

# Supple Figure S2 Nucleic acid metabolism with respect to uric acid production and xanthine oxidore-ductase (XOR)
Nucleic acid
Nucleoside
Adenosine
Inosine
Guanosine
Nucleoside
hypoxanthine
Adenine
Purine base
Xanthine
Guanine
Uric acid
Allantoin

## Slide 3
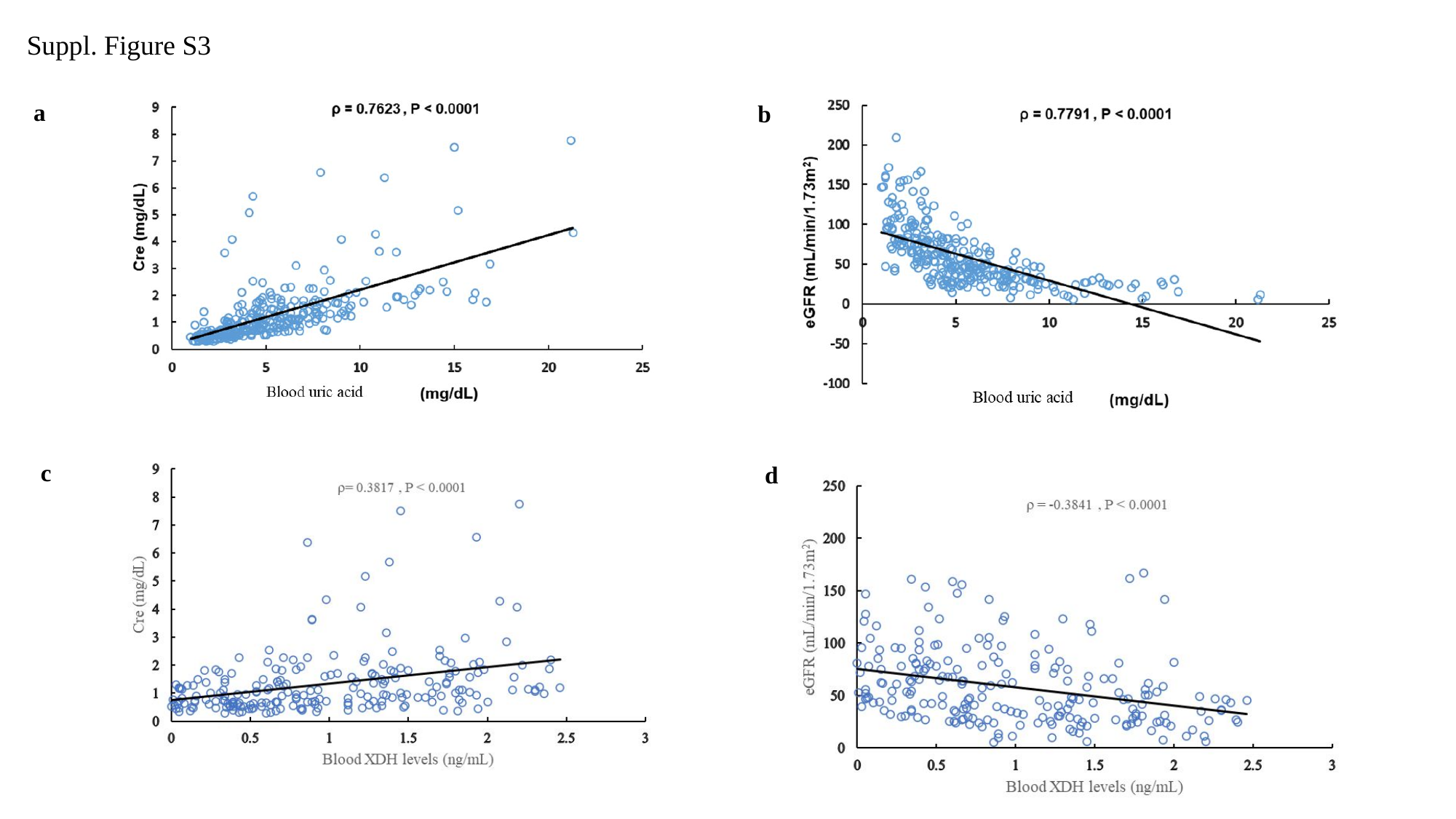

Suppl. Figure S3
a
b
c
d

## Slide 4
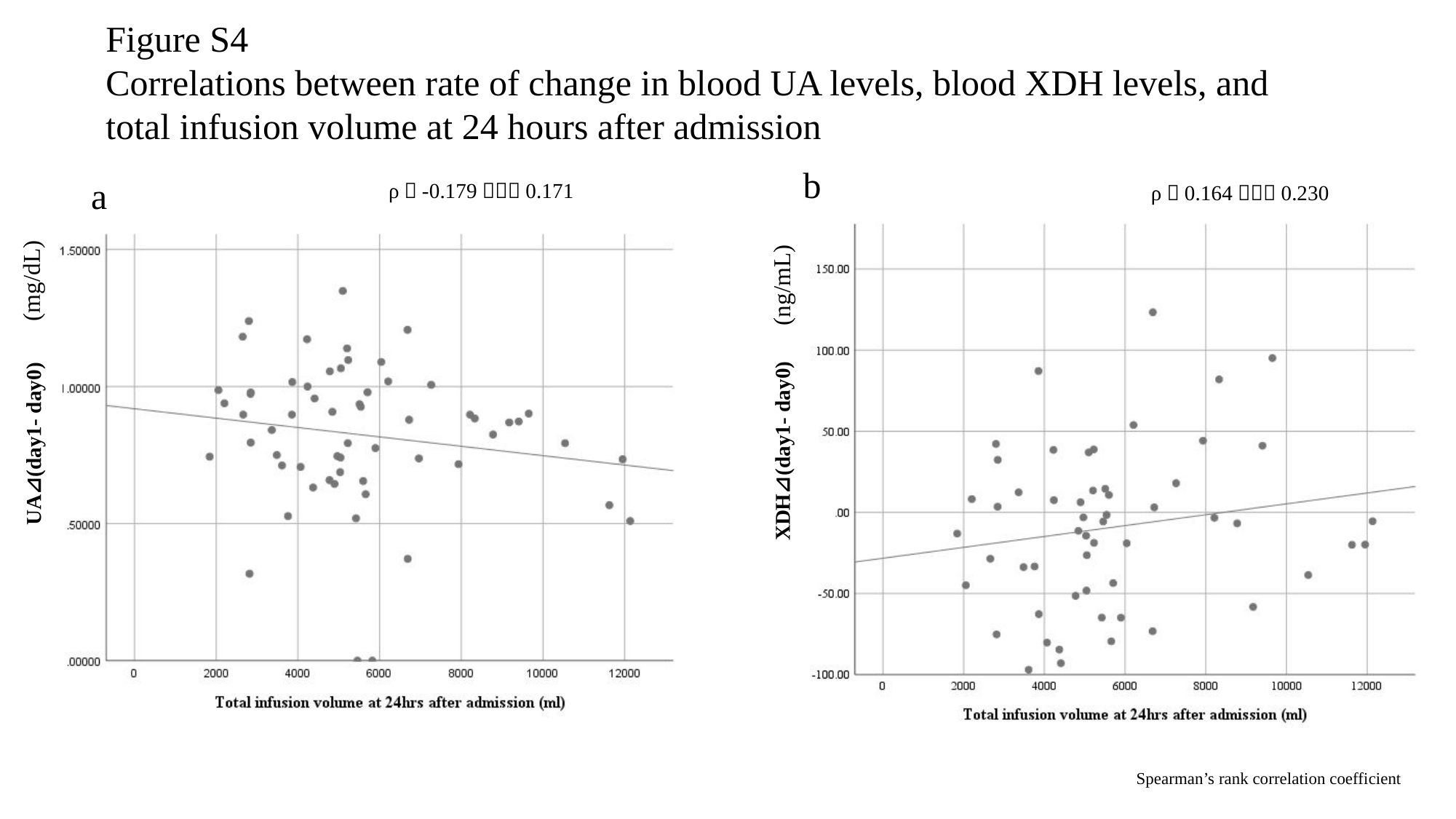

# Figure S4 Correlations between rate of change in blood UA levels, blood XDH levels, and total infusion volume at 24 hours after admission
b
a
ρ＝-0.179，ｐ＝0.171
ρ＝0.164，ｐ＝0.230
(mg/dL)
 (ng/mL)
 UA⊿(day1- day0)
XDH⊿(day1- day0)
Spearman’s rank correlation coefficient

## Slide 5
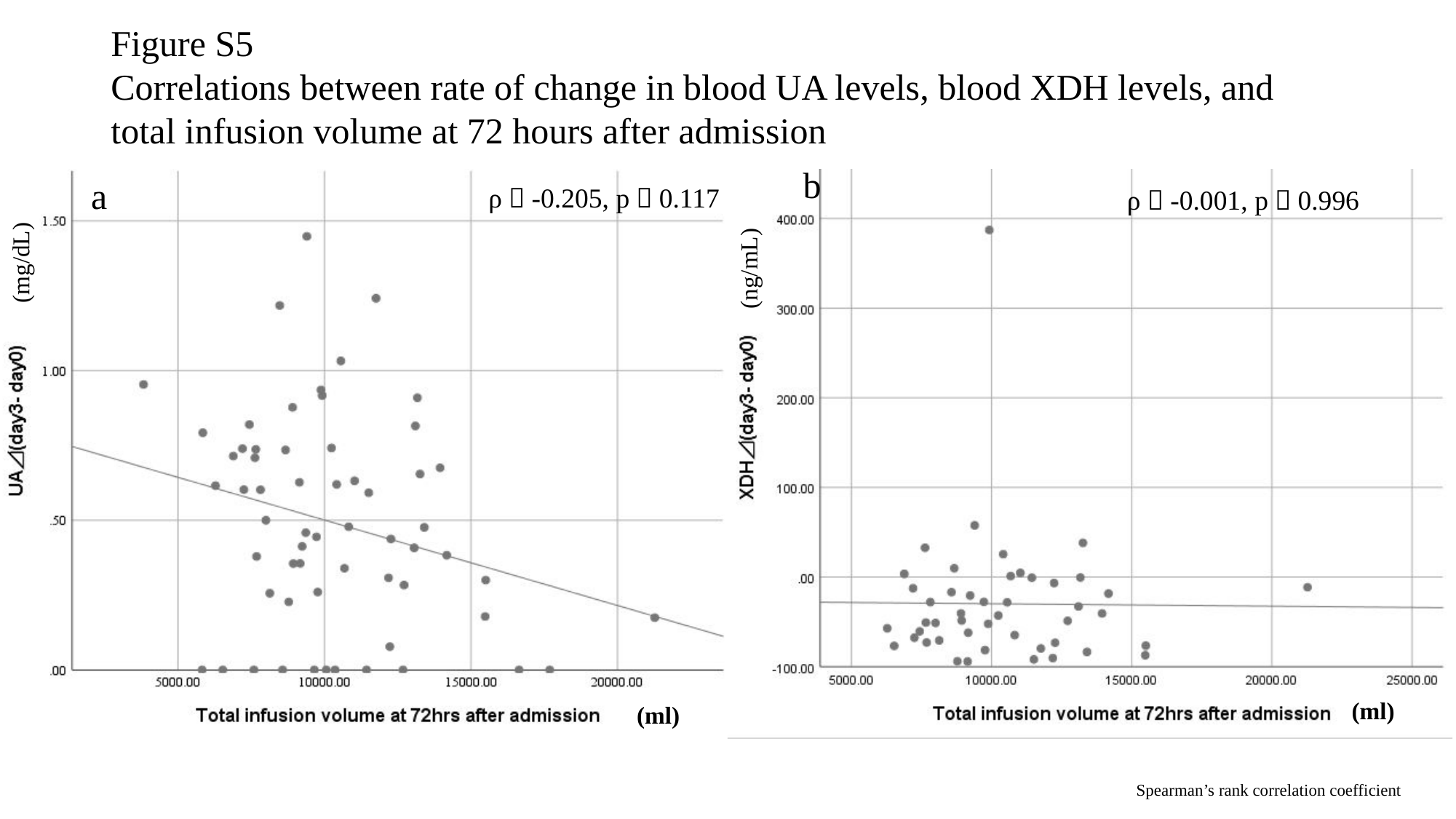

# Figure S5 Correlations between rate of change in blood UA levels, blood XDH levels, and total infusion volume at 72 hours after admission
b
a
ρ＝-0.205, p＝0.117
ρ＝-0.001, p＝0.996
(mg/dL)
 (ng/mL)
(ml)
(ml)
Spearman’s rank correlation coefficient

## Slide 6
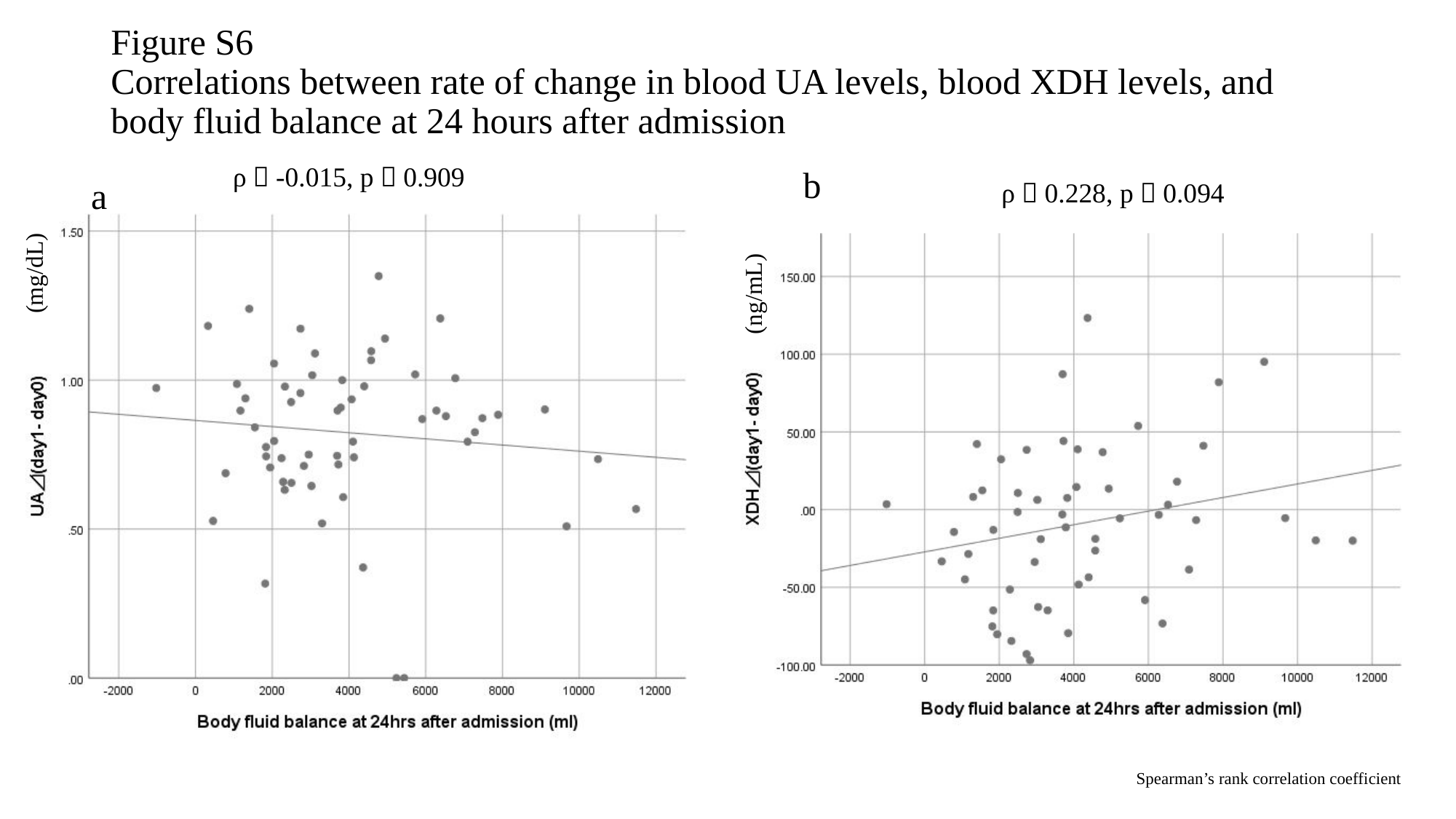

# Figure S6 Correlations between rate of change in blood UA levels, blood XDH levels, and body fluid balance at 24 hours after admission
ρ＝-0.015, p＝0.909
b
a
ρ＝0.228, p＝0.094
(mg/dL)
 (ng/mL)
Spearman’s rank correlation coefficient

## Slide 7
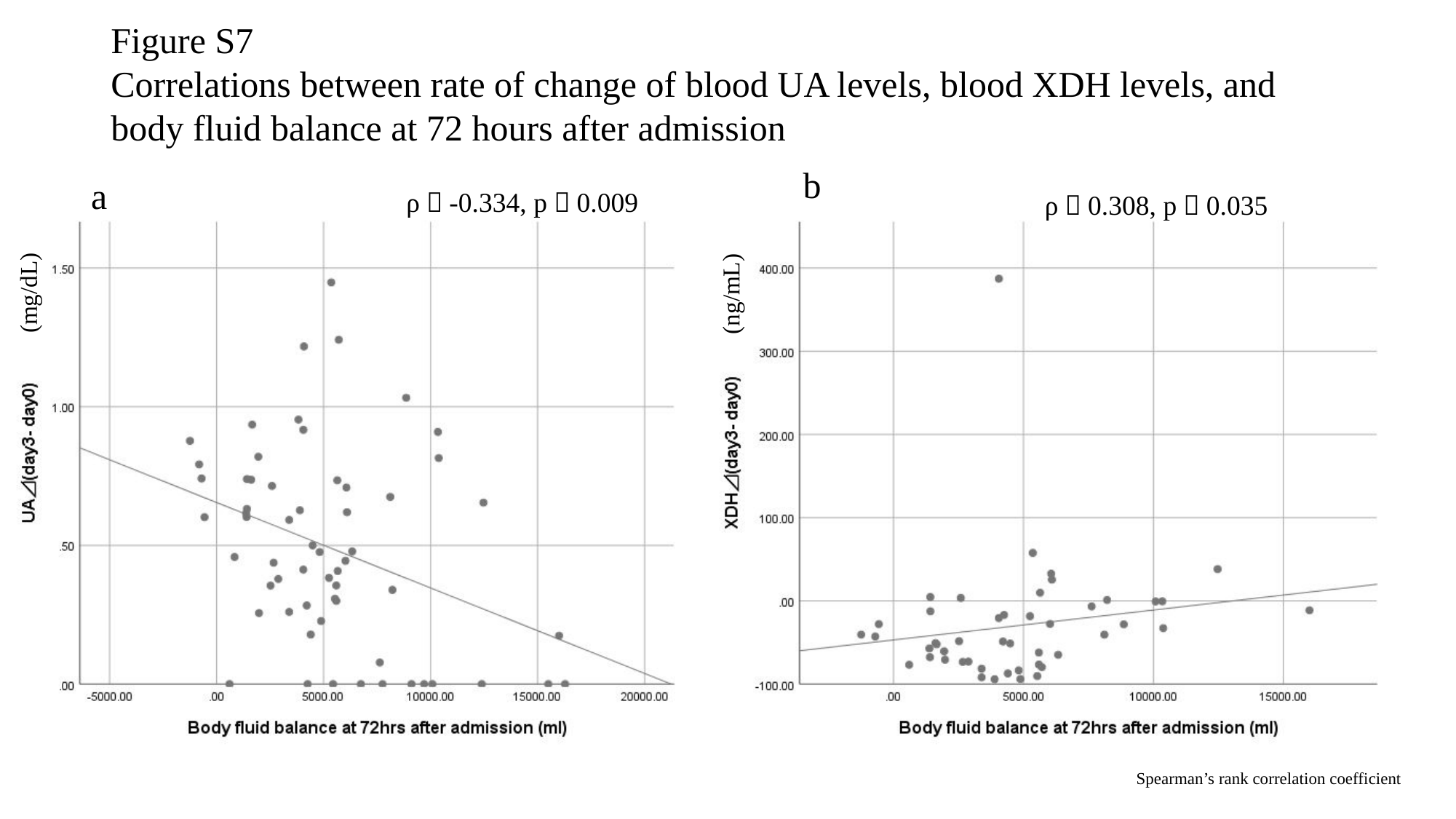

# Figure S7 Correlations between rate of change of blood UA levels, blood XDH levels, and body fluid balance at 72 hours after admission
b
a
ρ＝-0.334, p＝0.009
ρ＝0.308, p＝0.035
(mg/dL)
 (ng/mL)
Spearman’s rank correlation coefficient

## Slide 8
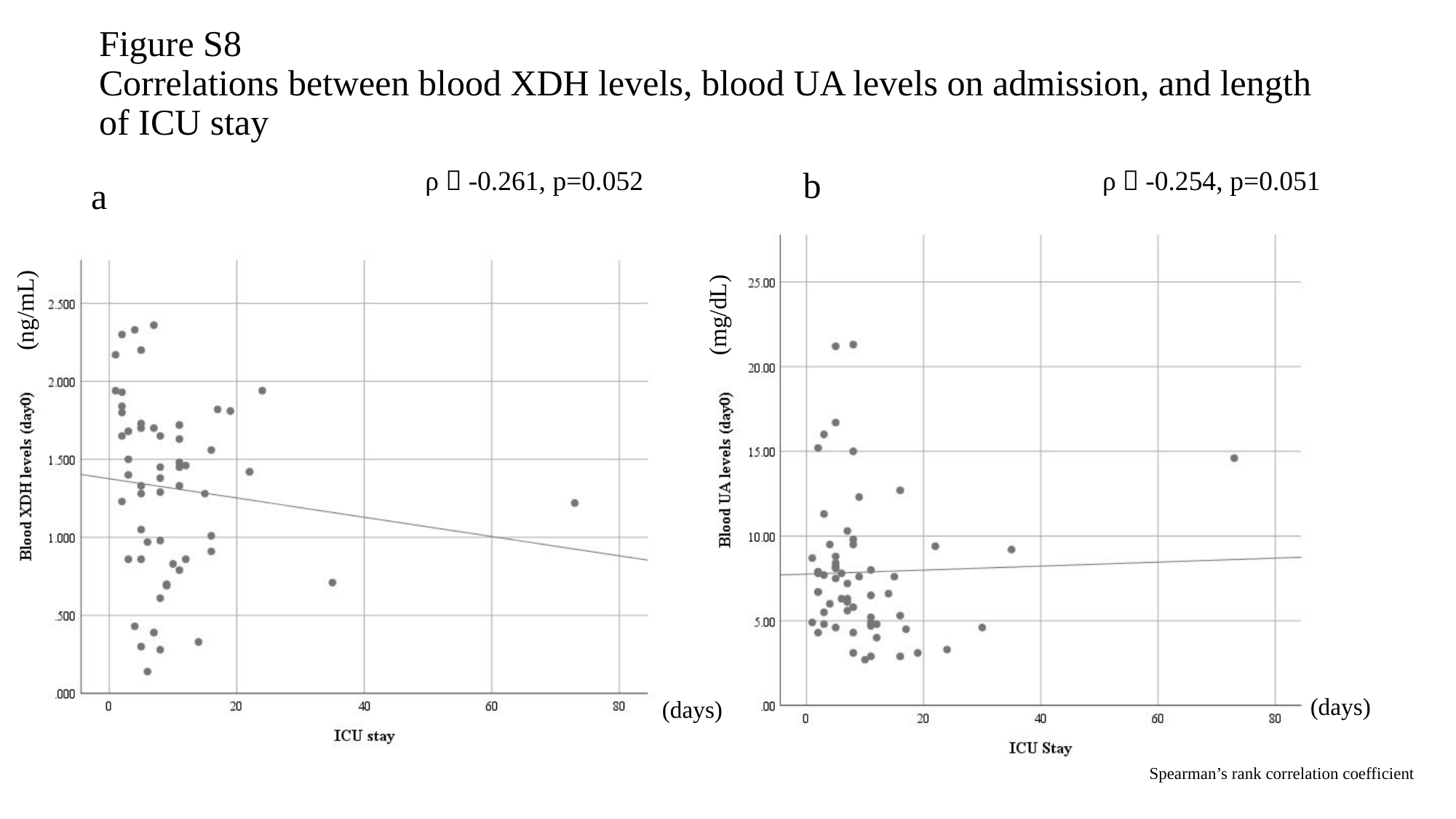

# Figure S8 Correlations between blood XDH levels, blood UA levels on admission, and length of ICU stay
b
ρ＝-0.254, p=0.051
ρ＝-0.261, p=0.052
a
 (ng/mL)
(mg/dL)
(days)
(days)
Spearman’s rank correlation coefficient

## Slide 9
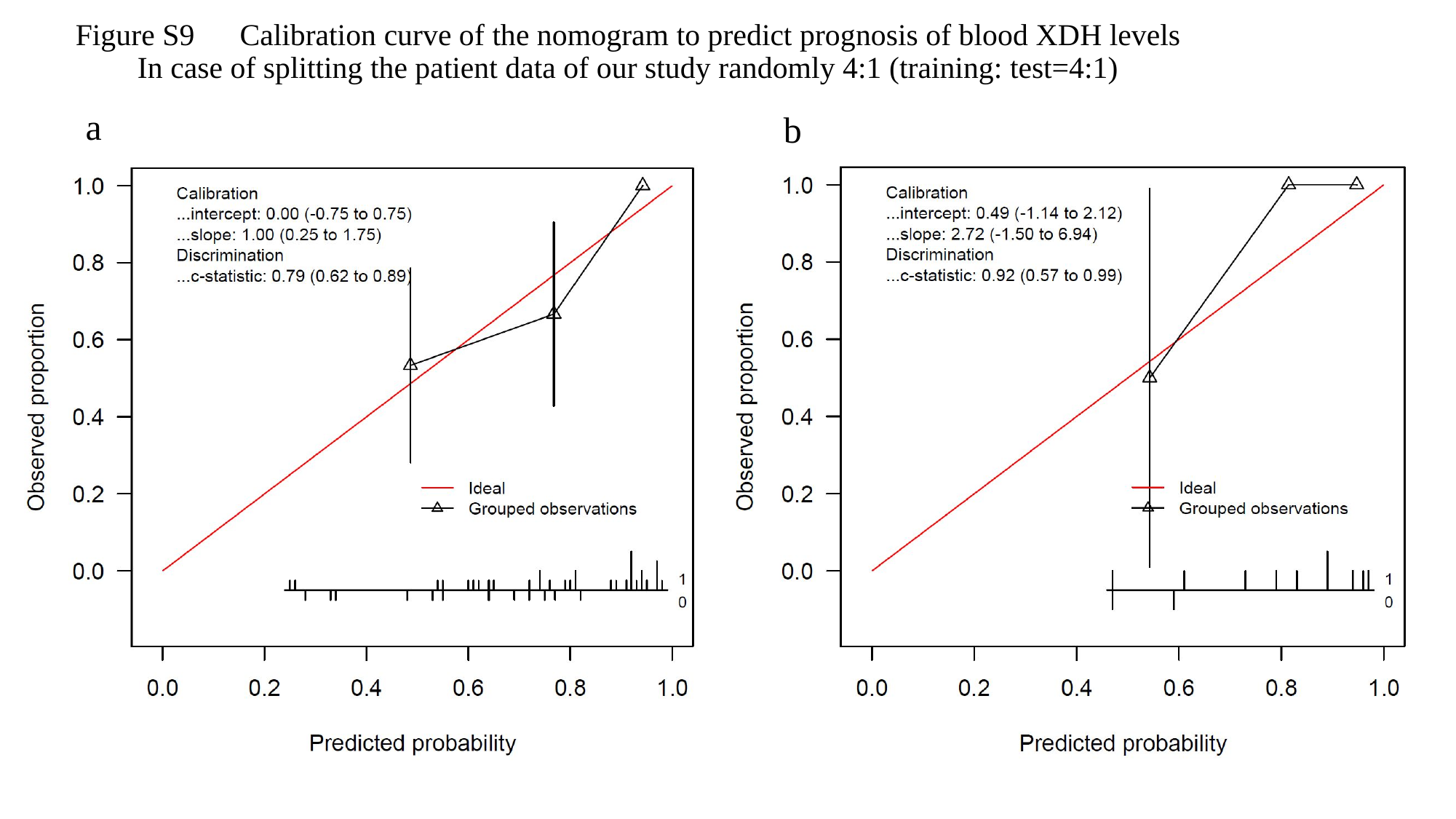

# Figure S9　Calibration curve of the nomogram to predict prognosis of blood XDH levels In case of splitting the patient data of our study randomly 4:1 (training: test=4:1)
a
b

## Slide 10
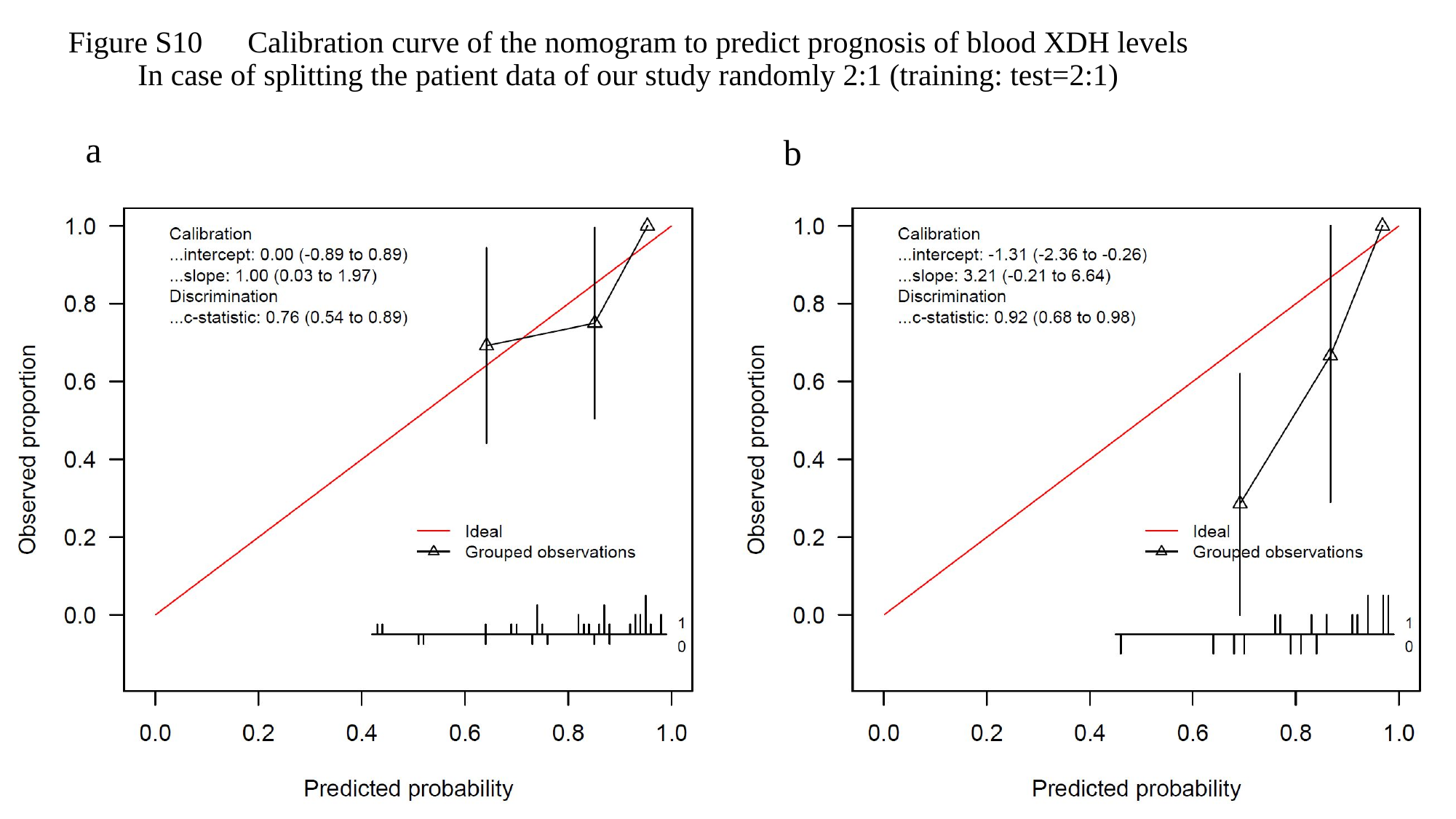

# Figure S10　Calibration curve of the nomogram to predict prognosis of blood XDH levels In case of splitting the patient data of our study randomly 2:1 (training: test=2:1)
a
b
