# Supplementary material for: Clinical Significance of Elevated Xanthine Dehydrogenase Levels and Hyperuricemia in Patients with Sepsis
Source: Int J Mol Sci. 2023 Sep 8;24(18):13857. doi: 10.3390/ijms241813857 (PMC10530551; doi:10.3390/ijms241813857)
Supplement: Supplementary file 1 [file ijms-24-13857-s001.zip › Supplementary Table S1 Validation of the prediction of blood XDH levels.pdf]

Supplementary Table S1 Validation of the prediction of blood XDH levels

Distribution	Values	Training cohort	Test cohort
In case of training: test=4:1＊	AUC (95%CI)	0.79 (0.62-0.89)	0.92 (0.57-0.99)
In case of training: test=2:1**	AUC (95%CI)	0.76 (0.54-0.89)	0.92 (0.68-0.98)

＊In total, patients enrolled in the first four-fifths of the study period were selected for the training group (n=44), and the remaining patients in the last fifth (n=12) were selected for the test group.

＊＊In total, patients enrolled in the first two-thirds of the study period were selected for the training group (n=37), and the remaining patients in the last third (n=19) were selected for the test group.

Our study cases of the data were split 4:1 or 2:1 (random assignment) and obtained AUC values and 95% confidence intervals for the training and test groups, respectively, using the bootstrap method. The upper part of the table below shows the results of the 4:1 split, while the lower part of the table shows the results of the 2:1 split.

In both distribution method studies, the 95% confidence intervals of the AUC were decreased in the training cohort and increased in the test cohort compared to the original predictive outcome of blood XDH levels (AUC=0.81).
